# A Rare Giant Dissecting Internal Carotid Artery Aneurysm With Middle and Fetal-Type Posterior Cerebral Artery Origins: A Case Report

**DOI:** 10.7759/cureus.105609

**Published:** 2026-03-21

**Authors:** Nirmal Sneha Kunnan Jimmy, Erekle Ekvtimishvili, Shota Ingorokva

**Affiliations:** 1 Surgery, Tbilisi State Medical University, Tbilisi, GEO; 2 Endovascular Neurosurgery, High Technology Medical Center, University Clinic, Tbilisi, GEO; 3 Neurosurgery, Tbilisi State Medical University, Tbilisi, GEO

**Keywords:** branch vessel preservation, dissecting aneurysm, endovascular treatment, flow diverter, giant aneurysm, internal carotid artery

## Abstract

Giant dissecting aneurysms of the internal carotid artery (ICA) >20 mm are rare and associated with risks of rupture, mass effect, and thromboembolism, particularly when branch vessels originate from the sac. A 46-year-old woman presented with headaches and blurred vision due to a giant dissecting aneurysm originating from the ophthalmic segment of the left internal carotid artery (ICA). Endovascular treatment using a flow-diverter stent (4.5×38 mm) was deployed from the left middle cerebral artery to the cavernous part of the ICA, with adjunctive balloon angioplasty for incomplete expansion due to vessel kinking. Immediate post-procedure angiography showed thrombosis with preserved branch perfusion. Symptoms resolved completely by one month, with dual antiplatelet therapy continued for six months and monotherapy for the next six months. This case highlights the efficacy of flow-diverter stents in complex anatomies, achieving rapid thrombosis and symptom relief.

## Introduction

Intracranial dissecting aneurysms are uncommon vascular lesions but are important to recognize because they can cause hemorrhage, ischemia, or be detected incidentally [1]. In 1994, Piepgras et al. reported a case of focal arterial wall dissection affecting a distal middle cerebral artery branch in a middle-aged woman, initially discovered incidentally during imaging evaluation for pulsatile tinnitus; serial angiography demonstrated progressive enlargement over six weeks, ultimately prompting intervention, and pathological analysis confirmed the dissecting nature of the lesion, leading the authors to propose that focal dissection be considered among the differential diagnoses of peripheral cerebral artery aneurysms [2].

With advances in imaging, unruptured intracranial aneurysms are being detected more often in clinical practice. Saccular (berry) aneurysms occur in about 1-2% of the population. Management of unruptured aneurysms requires weighing the risk of rupture against the risk of intervention (surgical clipping or endovascular treatment), taking into account aneurysm size, location, the patient’s medical and family history, and available treatment options with acceptable risk [3].

Giant dissecting aneurysms present unique anatomical and hemodynamic challenges that distinguish them from conventional saccular aneurysms. Unlike saccular aneurysms, which arise at arterial bifurcations with a defined neck, dissecting aneurysms result from pathological disruption of the arterial wall layers, producing fusiform or irregular dilatations without a true neck. This absent or poorly defined neck renders conventional clipping and coil embolization technically difficult or impossible [1]. The complexity is further magnified when critical branch vessels, such as the posterior communicating artery, middle cerebral artery, or a fetal-type posterior cerebral artery, originate directly from the aneurysmal sac rather than from the parent artery proper. In such configurations, any treatment strategy must reconcile the need for aneurysm exclusion with the imperative to preserve perfusion to essential branches, since their occlusion risks significant ischemic deficit. Lessons from complex vascular lesions with associated aneurysms in anatomically challenging locations have consistently shown that prioritizing early, definitive treatment of the aneurysm yields better outcomes, and that an experienced interdisciplinary team combining endovascular and surgical expertise is essential for minimizing complications in such cases [4]. The hemodynamic milieu within such an aneurysmal sac is additionally altered as follows: the sac functions as an arterial conduit for branch flow, creating competing inflow and pressure gradients that influence both the propensity for rupture and the likelihood of successful thrombosis following treatment [1].

The present case describes flow-diversion therapy using the FRED X (Aliso Viejo, CA: MicroVention, Inc.) device for a giant dissecting aneurysm of the internal carotid artery in which critical branch vessels (posterior communicating artery, fetal-type left posterior cerebral artery, and left middle cerebral artery) arose directly from the aneurysmal sac, with absent vertebrobasilar contribution to the left posterior cerebral artery (PCA). This rare configuration, with multiple essential branches originating from the sac, represents an exceptionally uncommon and complex morphology among intracranial aneurysms, significantly increasing treatment difficulty. Reporting such cases expands information on endovascular reconstruction in rare configurations that previously required high-risk surgery [5], highlighting flow diversion as a safer alternative that promotes thrombosis while preserving branch perfusion [6-8].

The configuration in which multiple critical vessels originate directly from an aneurysmal sac, rather than from the normal parent artery proximal or distal to the lesion, is particularly formidable. When a dominant vessel, such as a fetal-type posterior cerebral artery, supplying the entire posterior cerebral territory without vertebrobasilar contribution, originates from the sac, parent artery sacrifice or sac trapping would result in major posterior cerebral infarction. Similarly, coverage of such branches with a flow-diverting stent carries a non-negligible risk of branch occlusion as follows: the posterior communicating artery has a reported occlusion/narrowing rate of approximately 44% when covered by a flow diverter, though clinically significant ischemic complications remain acceptably low due to collateral compensation [9]. The fetal-type PCA variant further amplifies this risk, as its occlusion may not be adequately compensated by vertebrobasilar collaterals when the P1 segment is hypoplastic or absent [10]. Flow diversion was selected in our case precisely because it reconstructs the parent artery hemodynamically, redirecting flow away from the sac while preserving perfusion through the device’s interstices to incorporated branches, rather than sacrificing or trapping any vessel [6-8].

Flow diversion has emerged as a highly effective endovascular strategy for treating large and giant intracranial aneurysms, including complex cases with incorporated or covered branches [6,7,11,12]. Recent multicenter experiences with the FRED X flow diverter, featuring antithrombotic surface technology, have demonstrated high procedural feasibility, low thrombotic complication rates, and adequate mid-term occlusion outcomes [11,12]. When flow-diverting stents cover critical branches, such as the ophthalmic artery, posterior communicating artery, anterior choroidal artery, or anterior cerebral artery, aneurysm obliteration rates remain satisfactory (>70% at 12 months), though branch occlusion risks vary by vessel (e.g., higher for anterior cerebral artery {ACA} or posterior communicating artery {PCoA}, lower for ophthalmic artery {OA}, or anterior choroidal artery {AChA}), with overall low complication incidence (<5%) [9]. Subanalyses from international registries further support the safety and effectiveness of flow diversion for aneurysms with incorporated branches, highlighting the influence of branch origin on occlusion dynamics [13]. Comparative systematic reviews indicate that flow diversion achieves significantly lower recurrence rates (8% vs. 27%) and a trend toward higher rates of complete occlusion compared with coiling for large and giant aneurysms, with similar overall safety profiles despite a modest increase in hemorrhagic risk [14].

## Case presentation

**Figure 1:**
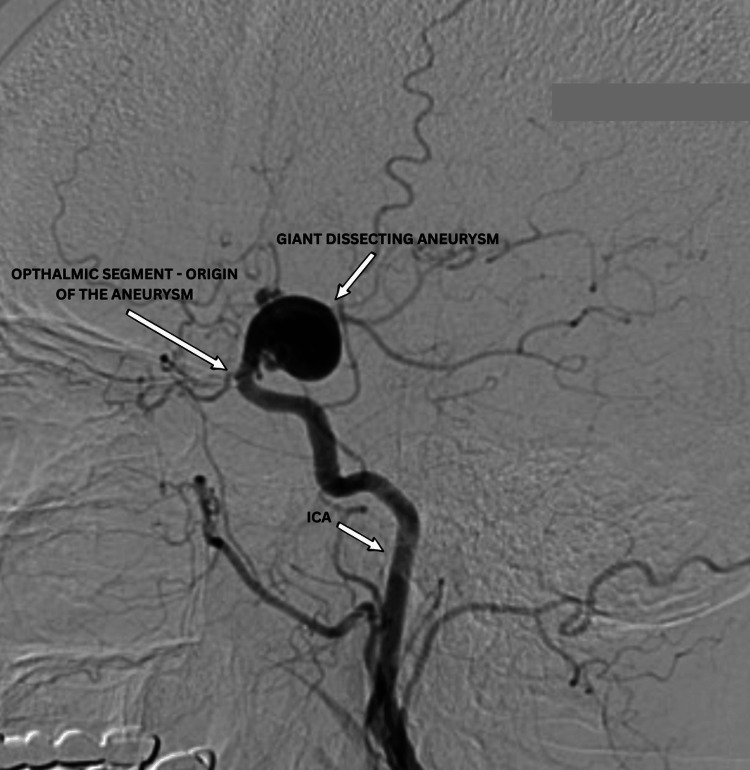
Pre-operative DSA (lateral view) showing a giant dissecting aneurysm of the ICA arising from the ophthalmic segment. DSA: digital subtraction angiography; ICA: internal carotid artery

A 46-year-old woman presented with worsening intermittent headaches and blurred vision for the past three months, with no prior medical history, chronic illnesses, or substance use. This was her first medical consultation for these symptoms. After evaluation by a neurologist, magnetic resonance imaging (MRI) was directly performed and revealed a large aneurysm in the left internal carotid artery (ICA) without hemorrhage (Figure 1).

**Figure 2:**
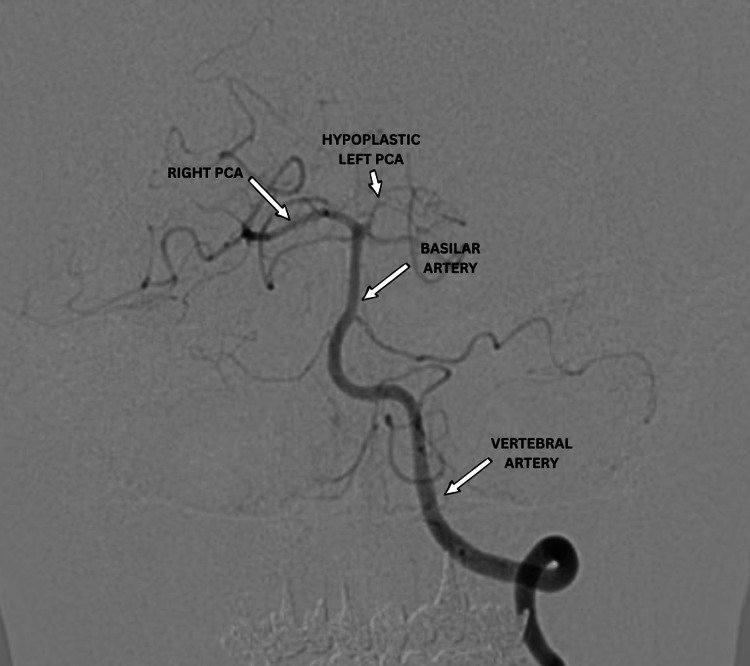
Imaging of the vertebrobasilar system demonstrates an asymmetric hypoplastic left P1 segment and a dominant left fetal-type PCA supplied by the carotid system, while the right PCA and the basilar and vertebral arteries appear normal. PCA: posterior cerebral artery

**Figure 3:**
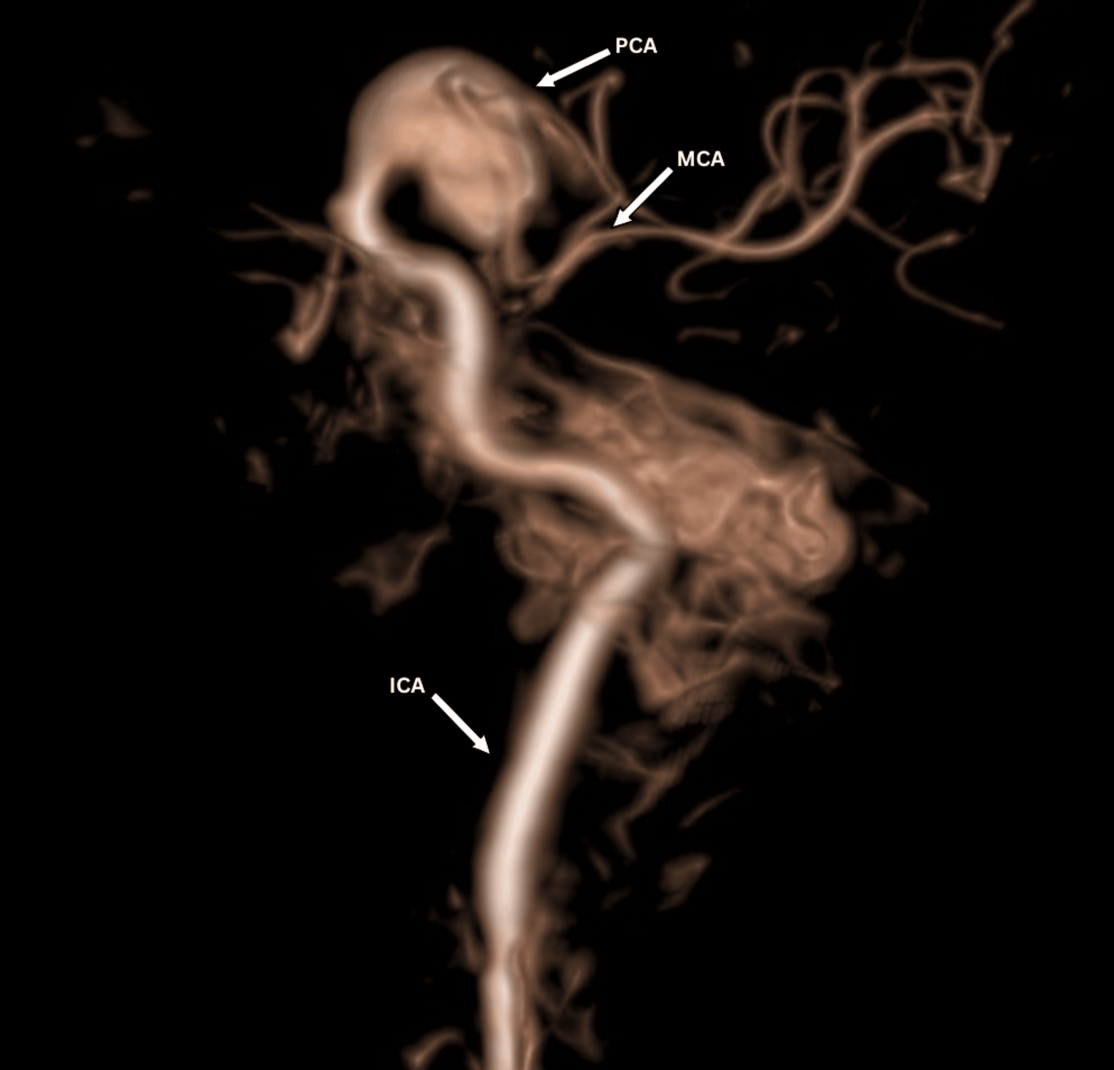
Three-dimensional view of the aneurysm showing PCA and MCA arising from the aneurysmal sac. PCA: posterior cerebral artery; MCA: middle cerebral artery; ICA: internal carotid artery

Digital subtraction angiography (DSA) confirmed a giant dissecting aneurysm (>20 mm) originating from the ophthalmic segment of the left ICA. The posterior communicating artery, followed by the fetal-type left posterior cerebral artery (PCA), and the left middle cerebral artery (MCA) arose directly from the aneurysmal sac. The left PCA was not seen from the vertebrobasilar system (Figure 2). Three-dimensional imaging was performed and is shown in Figure 3.

**Figure 4:**
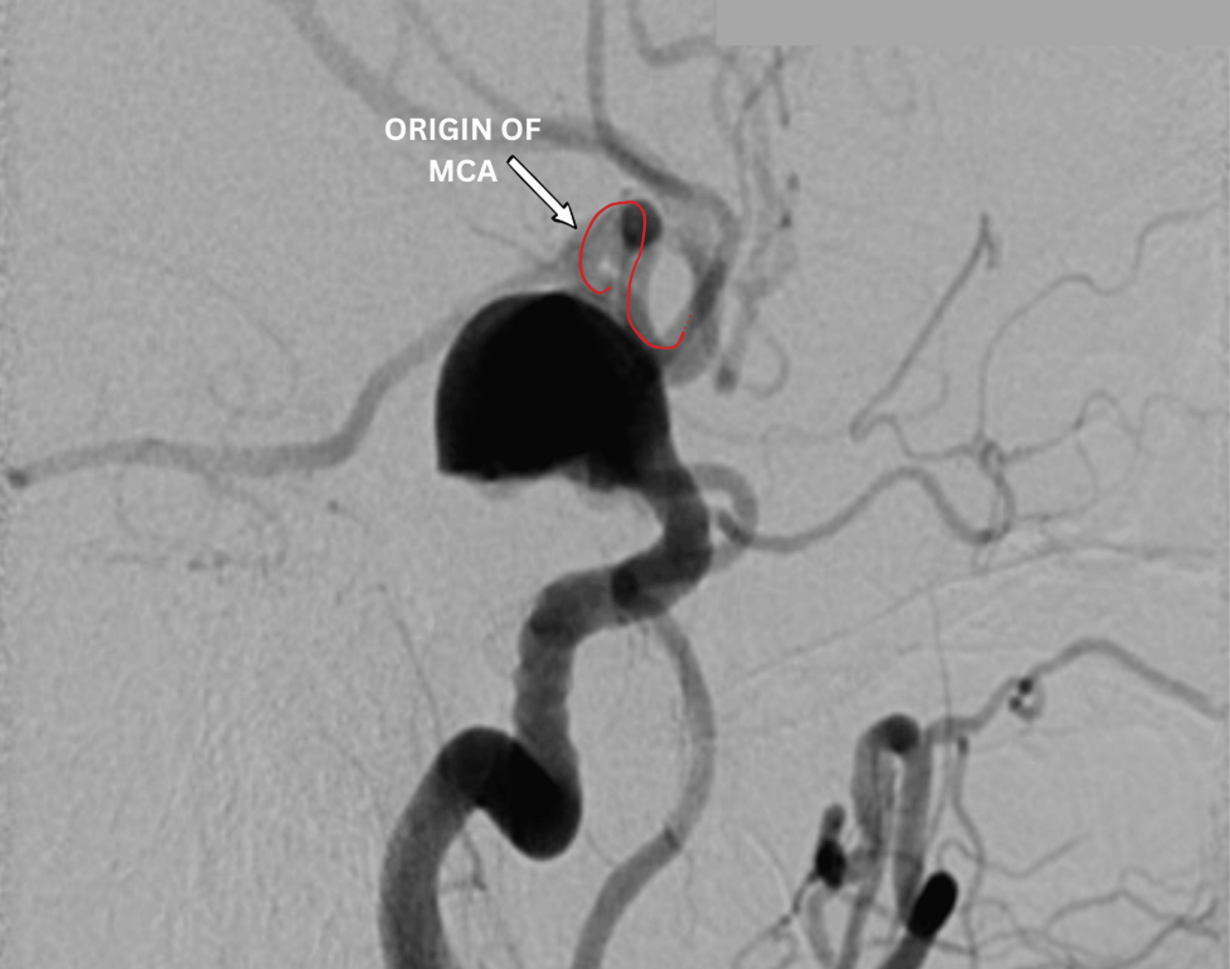
Peri-operative DSA showing the origin of the MCA from the dissecting giant aneurysm. DSA: digital subtraction angiography; MCA: middle cerebral artery

**Figure 5:**
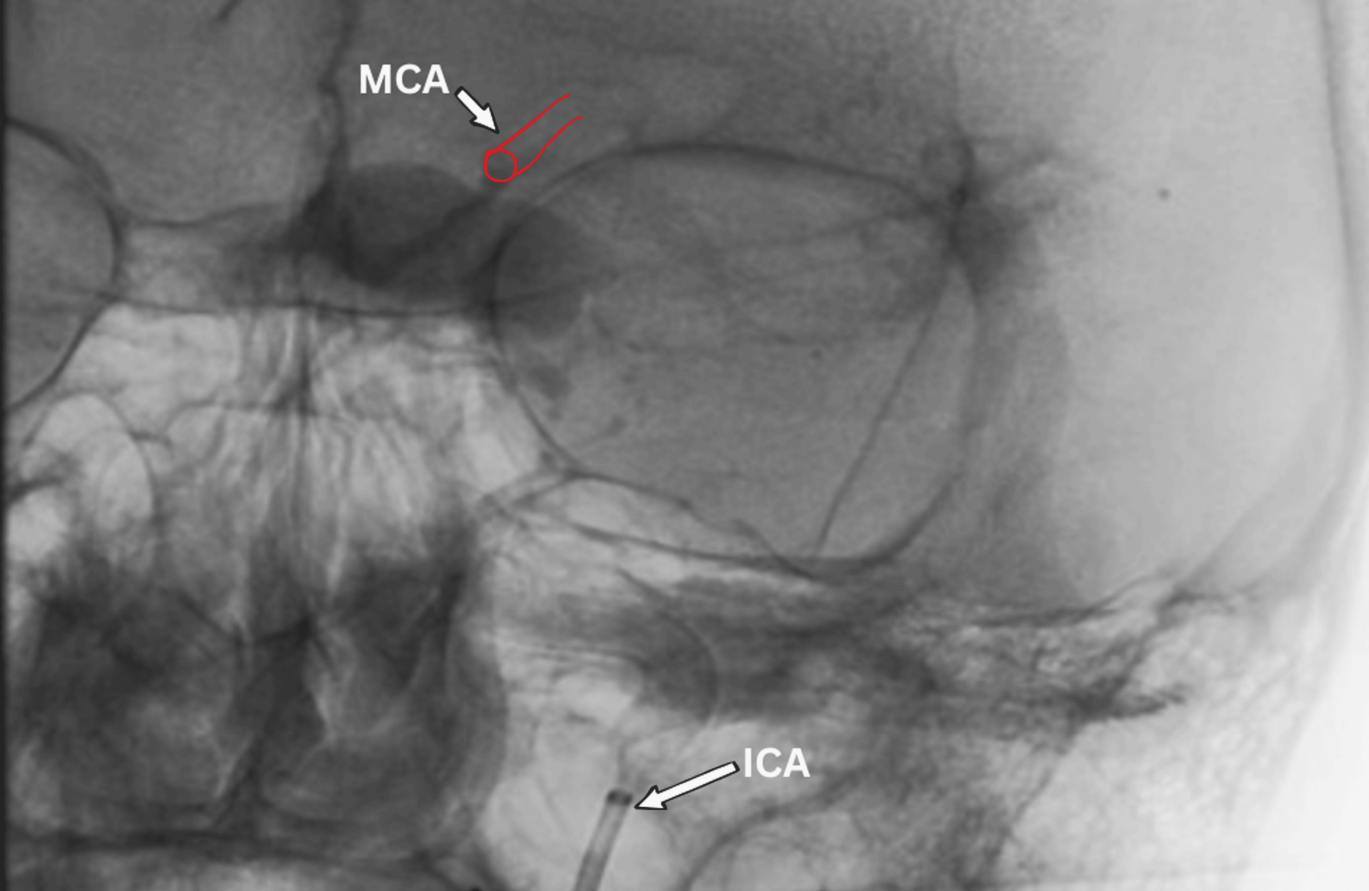
Working projection peri-operative DSA showing advancement of microcatheter from ICA into MCA originating from dissecting giant aneurysm. DSA: digital subtraction angiography; MCA: middle cerebral artery; ICA: internal carotid artery

**Figure 6:**
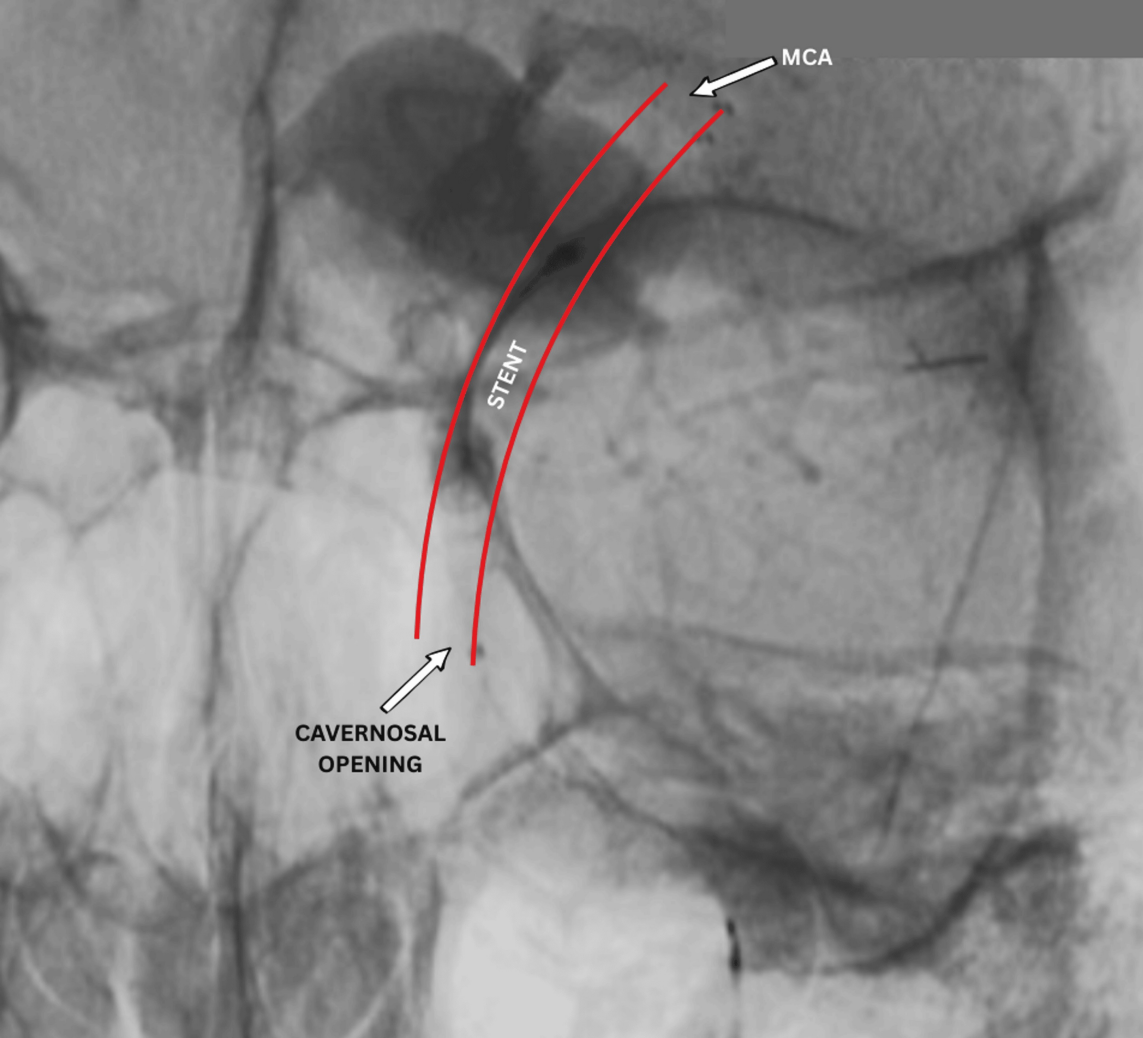
Peri-operative DSA demonstrating opening of the flow-diverting stent (red outlines) from the cavernous segment of the ICA to the MCA (white arrow). DSA: digital subtraction angiography; MCA: middle cerebral artery; ICA: internal carotid artery

**Figure 7:**
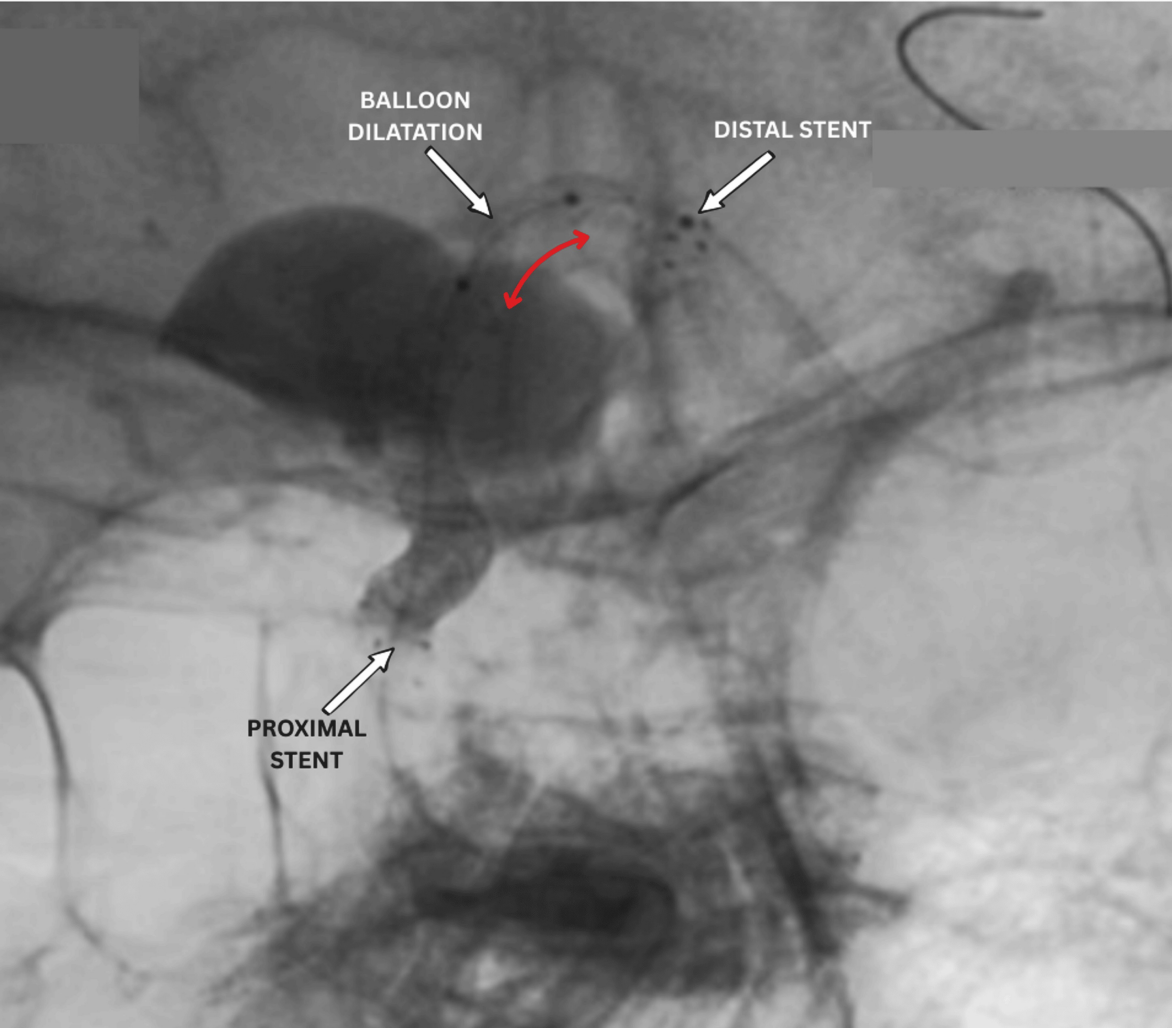
Peri-operative DSA showing balloon dilatation (red arrow) being performed within the flow-diverting stent, with proximal stent (white arrow) in the cavernous ICA segment and distal stent (white arrow) extending into the MCA. DSA: digital subtraction angiography; MCA: middle cerebral artery; ICA: internal carotid artery

Due to escalating symptoms and risks of rupture or thromboembolism, intervention was indicated. Microsurgical clipping was deemed too risky due to the aneurysm’s size and location, which increased intra-operative and post-operative complication risks. An endovascular approach using a flow-diverter stent (in our case, FRED X) was chosen to redirect blood flow and induce sac thrombosis. The procedure involved angiographic mapping to locate the MCA’s origin (Figure 4). A long sheath destination 6F was navigated into the left ICA. Then, through an intermediate guiding catheter, a FARGO 6F microcatheter (Headway 27) over the micro guidewire Traxcess 14 was navigated distally into the MCA (Figure 5), followed by deployment of a flow-diverter stent (4.5 mm diameter, 38 mm length) from the left MCA to the cavernous part of the ICA (Figure 6). Incomplete stent expansion at the MCA origin, likely due to vessel kinking, was observed. This necessitated balloon catheter (Scepter XC 4 mm × 11 mm tube) dilatation to achieve full apposition (Figure 7).

**Figure 8:**
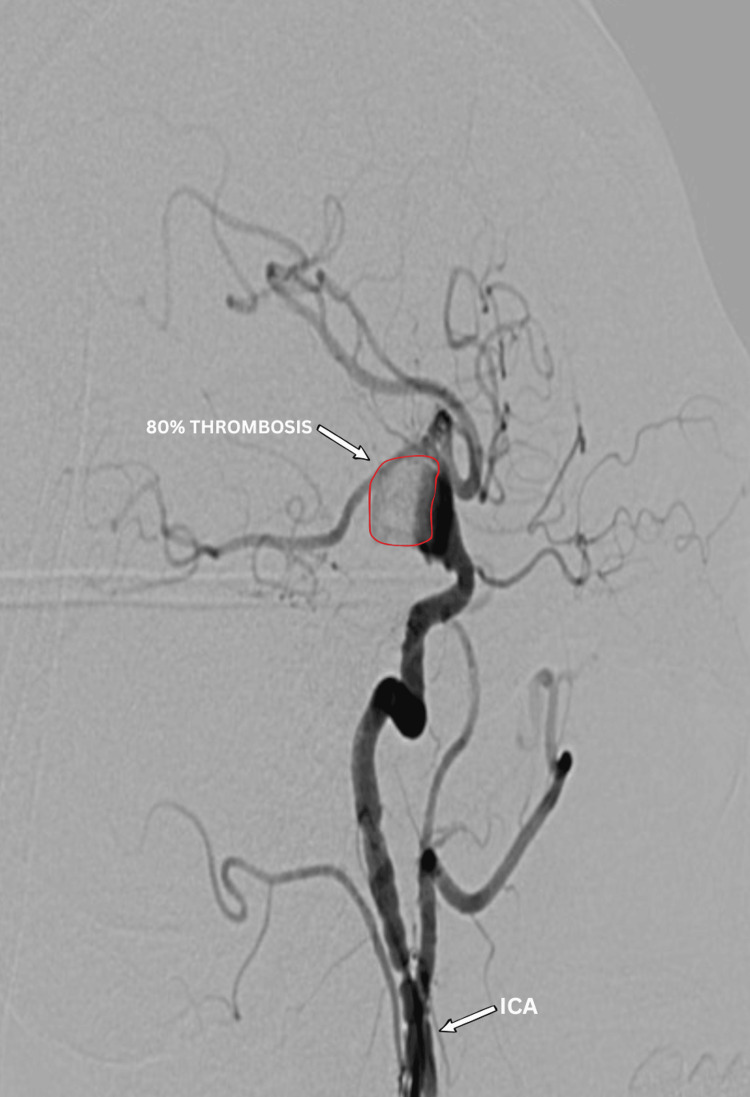
Post-operative DSA (lateral view) showing bulk thrombosis (≈75%, annotated) of the giant dissecting aneurysm with reduced residual contrast filling and preserved flow through the stented parent vessel (ICA to MCA). DSA: digital subtraction angiography; MCA: middle cerebral artery; ICA: internal carotid artery

**Figure 9:**
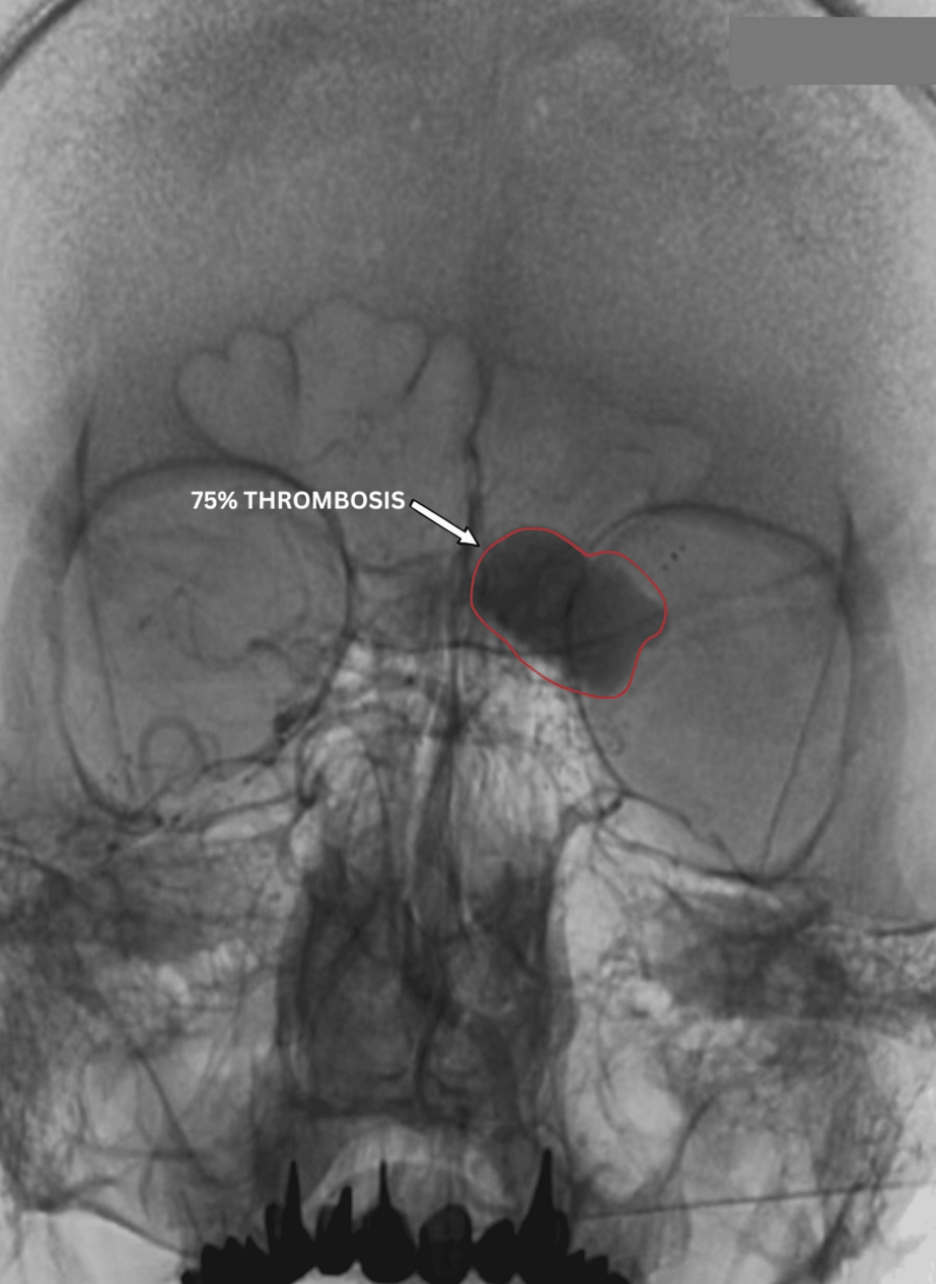
Post-operative DSA in direct (anteroposterior) view showing bulk thrombosis (≈75%, annotated with arrow and outline) of the aneurysm with preserved patency of the flow-diverted parent vessel (ICA to MCA). DSA: digital subtraction angiography; MCA: middle cerebral artery; ICA: internal carotid artery

**Figure 10:**
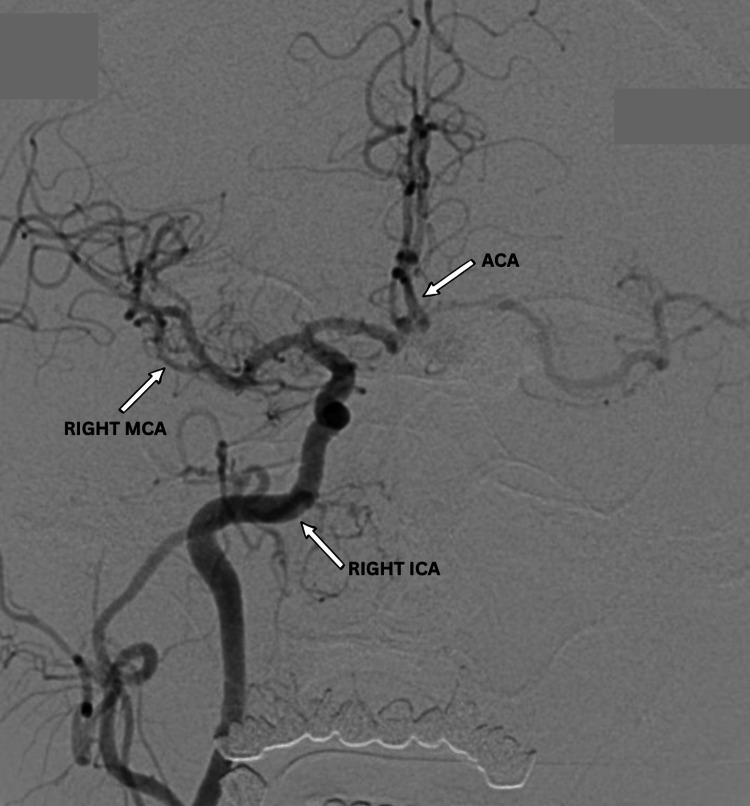
Post-operative DSA showing collateral filling of the right MCA (white arrow) through the ACA, with preserved distal perfusion and exclusion of the giant dissecting aneurysmal sac following flow-diverting stent placement (right ICA annotated). DSA: digital subtraction angiography; MCA: middle cerebral artery; ICA: internal carotid artery; ACA: anterior cerebral artery

Post-procedural angiography showed 70-75% aneurysm thrombosis, with preserved MCA and PCA perfusion (Figures 8, 9). No aneurysm filling was observed from the contralateral side via the anterior communicating artery (Figure 10). Dual antiplatelet therapy included ticagrelor 90 mg twice daily and aspirin 100 mg daily, started five days pre-operatively, with a 300 mg aspirin loading dose. This regimen continued for six months post-procedure, followed by aspirin 100 mg daily alone for another six months. The patient was discharged after three days without complications, with headaches and visual symptoms significantly reduced. Complete symptom resolution was noted at the one-month follow-up.

## Discussion

Before endovascular techniques, management depended mainly on microsurgical methods such as clipping, trapping, or bypass. These were technically demanding for proximal internal carotid lesions and carried substantial procedural risk, with phase-based complications involving exposure, vascular control, aneurysm dissection, clip application, and verification; high-volume centers and surgeon experience correlate with fewer adverse events, and checklists/standardized bailout strategies are recommended to reduce variance [5].

Flow-diversion therapy provides a reconstructive alternative. As demonstrated by Lylyk et al., a metal-mesh device like the pipeline embolization device redirects hemodynamic flow away from the aneurysm, promoting gradual thrombosis while maintaining distal perfusion in wide-necked, large, giant, non-saccular, or recurrent aneurysms, with 95% of aneurysms achieving complete occlusion by 12 months with no major complications and only rare transient symptom exacerbations [6]. Becske et al. confirmed safety and effectiveness in large or giant wide-necked unruptured internal carotid aneurysms unsuitable for coiling or clipping, with near-complete technical success, showing over 73% complete occlusion at 180 days [7].

Later analyses defined its role in dissecting aneurysms. Cagnazzo et al. summarized that flow-diverter implantation is effective for endovascular reconstruction of dissecting/fusiform and blister aneurysms, diverting flow to promote progressive thrombosis despite challenges like thin walls or absent true necks [8].

The uniqueness of our case lies in the exceptionally rare configuration of a giant dissecting aneurysm (>20 mm) in the ophthalmic segment of the left ICA, where the posterior communicating artery, the fetal-type left PCA, and the left middle cerebral artery (MCA) arose directly from the aneurysmal sac, with the left PCA not visible from the vertebrobasilar system. Such presentations with multiple key branches originating directly from the sac are exceptionally uncommon, adding substantial complexity to treatment (rarity implied in complex dissecting/giant lesions [2,6-8]; branch incorporation challenges [9,13]).

Flow-diverter stents like the FRED X redirect blood along the main vessel, causing gradual clotting inside the aneurysm while keeping blood flow to nearby branches [9,11]. In this case, post-procedure angiography showed ~70% thrombosis, preserved MCA and PCA flow, and no filling from the opposite side via the anterior communicating artery. Precise deployment with balloon dilatation achieved full apposition despite vessel kinking, highlighting technical feasibility [11,12].

This anatomy amplified treatment challenges, as traditional microsurgical clipping posed elevated risks of intra-operative rupture, thromboembolism, or branch occlusion due to the aneurysm’s size, location, and direct branch involvement. Surgical approaches for proximal ICA lesions carry significant procedural risks related to exposure, vascular control, dissection, and clip verification [5].

Evidence supports preserved branch patency in most cases when branches are covered, with low related ischemic complications; patency is maintained in ophthalmic, anterior choroidal, posterior communicating, and MCA-M2 branches in many patients, though occlusion/narrowing risks are higher in ACA, PComA, and MCA-M2 but with acceptable clinical rates due to collateral compensation [9]. Branch origin influences occlusion outcomes, with dome-origin branches associated with lower rates of complete occlusion, yet flow diversion maintains a favorable safety profile with low rates of stroke or branch occlusion [13].

Compared with previously reported cases of dissecting aneurysms with incorporated branches, our case presented a uniquely compound configuration in which three major branch vessels, the PComA, the fetal-type PCA, and the MCA, all arose from a single giant sac. In the literature, most reported cases of branch incorporation involve a single vessel, typically the posterior inferior cerebellar artery in vertebral artery dissections, or the ophthalmic artery in ICA aneurysms, where treatment decisions largely follow established protocols [1].

Cases in which both the MCA and a fetal-type PCA simultaneously originate from the same ICA sac have rarely been documented, and no standardized management algorithm exists for this configuration. Covering branches with a flow diverter can narrow or block them - about 39% for MCA-M2 and 44% for PComA. However, serious ischemic complications are usually under 5% because collateral blood flow compensates [9]. The absence of vertebrobasilar supply to the left PCA in our patient, with the entire posterior cerebral territory depending on the fetal-type PCA routed through the aneurysmal sac, significantly elevated the ischemic consequence of branch occlusion and required heightened vigilance during stent deployment and post-operative monitoring. Furthermore, prior series have identified fetal-type PCA anatomy as a factor associated with reduced flow diverter efficacy, as the high-flow demand of the fetal vessel may sustain competing inflow into the sac, impeding thrombosis [10].

Consistent with the principle established in the management of complex vascular lesions with associated aneurysms in high-risk anatomical locations, where early, targeted treatment of the aneurysm by an experienced interdisciplinary team has been shown to reduce complications and improve outcomes, our approach prioritized aneurysm reconstruction over any adjunctive maneuver that might jeopardize branch perfusion [4]. The successful preservation of both MCA and PCA perfusion following FRED X deployment in our case underscores the reconstructive advantage of flow diversion over deconstructive strategies in such high-stakes anatomical configurations [8,9,13].

For large and giant aneurysms, flow diversion achieves more reliable exclusion and lower recurrence than conventional coiling. A systematic review and meta-analysis found that flow diversion significantly reduced recurrence (8% vs. 27% for coiling) and trended toward higher complete occlusion, with comparable overall safety but modestly increased hemorrhagic risk [14].

Dual antiplatelet regimens (ticagrelor and aspirin in our case) complemented the approach to minimize thromboembolic events, yielding swift symptom relief, no complications, and discharge after three days. Collectively, these observations demonstrate that flow-diversion devices enable safe and effective reconstruction of arteries affected by dissecting or complex aneurysms with branch incorporation, facilitating thrombosis while preserving physiological circulation and representing a major advance over traditional surgical treatment [5-9,11-14].

## Conclusions

To conclude, giant dissecting ICA aneurysms are rare, with branch vessel incorporation from the sac adding significant complexity. Presentation mirrors typical aneurysm symptoms like headache and visual deficits, predominantly in middle-aged women. While surgical options carry high morbidity, endovascular flow diversion offers high occlusion rates and low complication rates, as demonstrated in our case with rapid thrombosis and symptom resolution.
